# Benefits of antifungal therapy in asthma patients with airway mycosis: A retrospective cohort analysis

**DOI:** 10.1002/iid3.215

**Published:** 2018-03-25

**Authors:** Evan Li, Chu‐Lin Tsai, Zahida K. Maskatia, Ekta Kakkar, Paul Porter, Roger D. Rossen, Sarah Perusich, John M. Knight, Farrah Kheradmand, David B. Corry

**Affiliations:** ^1^ Department of Medicine Baylor College of Medicine Houston Texas USA; ^2^ Department of Emergency Medicine National Taiwan University Hospital Taipei Taiwan; ^3^ Department of Pathology and Immunology Baylor College of Medicine Houston Texas USA; ^4^ Department of Biology of Inflammation Center Baylor College of Medicine Houston Texas USA; ^5^ Michael E. DeBakey VA Center for Translational Research on Inflammatory Diseases Houston Texas USA

**Keywords:** Airway mycosis, antifungal, asthma

## Abstract

**Introduction:**

Fungal airway infection (airway mycosis) is increasingly recognized as a cause of asthma and related disorders. However, prior controlled studies of patients treated with antifungal antibiotics have produced conflicting results. Our objective is to measure the effect of antifungal therapy in moderate to severe adult asthmatics with positive fungal sputum cultures in a single center referral‐based academic practice.

**Methods:**

We retrospectively evaluated 41 patients with asthma and culture‐proven airway mycosis treated with either terbinafine, fluconazole, itraconazole, voriconazole, or posaconazole for 4 to >12 weeks together with standard bronchodilator and anti‐inflammatory agents. Asthma control (1 = very poorly controlled; 2 = not well controlled; and 3 = well controlled), peak expiratory flow rates (PEFR), serum total IgE, and absolute blood eosinophil counts before and after antifungal therapy were assessed. In comparison, we also studied nine patients with airway mycosis and moderate to severe asthma who received standard therapy but no antifungals.

**Results:**

Treatment with azole‐based and allylamine antifungals was associated with improved asthma control (mean change in asthma control 1.72–2.25; *p* = 0.004), increased PEFR (69.4% predicted to 79.3% predicted, *p* = 0.0011) and markedly reduced serum IgE levels (1,075 kU/L to 463 kU/L, *p* = 0.0005) and blood eosinophil counts (Mean absolute count 530–275, *p* = 0.0095). Reduction in symptoms, medication use, and relapse rates decreased as duration of therapy increased. Asthmatics on standard therapy who did not receive antifungals showed no improvement in asthma symptoms or PEFR. Antifungals were usually well tolerated, but discontinuation (12.2%) and relapse (50%) rates were relatively high.

**Conclusion:**

Antifungals help control symptoms in a subset of asthmatics with culture‐proven airway mycosis. Additional randomized clinical trials are warranted to extend and validate these findings.

## Introduction

The hygiene hypothesis suggests that allergic diseases such as asthma, and chronic, as well as seasonal atopic rhinosinusitis (CRS) may be a consequence of maladaptive immune reactions to microorganisms and other environmental agents beginning in childhood 1. The presumed immunologic basis for these conditions provides the rationale for treatment of these disorders with agents that attenuate inflammation and suppress immune responses. While these therapies help to control symptoms, they have failed to reduce the increasing prevalence of allergic diseases in the United States 2.

Its diverse clinical presentations suggest that asthma is a manifestation of many distinct pathophysiologies 3. Proteinases derived from bacteria and fungi, as well as plants, have long been implicated in the pathogenesis of occupational asthma. For example, it is well established that proteinases derived from *Bacillus subtilis* (subtilisins) when added to laundry detergents can promote severe, but reversible asthma 4, 5. Indeed a large variety of exogenous and endogenous proteinases can initiate or at least help to promote allergic and asthmatic reactions in humans and experimental systems 6, 7, 8, 9, 10, 11, 12, 13.

The most intensively studied allergenic proteinases are those derived from fungi. Given intranasally to mice, the major secreted proteinases from the fungus, *Aspergillus oryzae* are alone sufficient to induce major features of asthma, including T helper type 2 (T_H_2) cell recruitment to lungs, airway hyperresponsiveness, goblet cell metaplasia, airway eosinophilia, and elevated serum IgE in a manner that requires intact proteinase activity 13. *A. oryzae* proteinases drive dendritic cell maturation in a way that favors T_H_2 cell expansion and the neutralization of cytokines such as IL‐12 that antagonize T_H_2 responses 14. These and similar proteinases also recruit innate lymphoid cells type 2 (ILC2) that promote allergic responses when acting together with T_H_2 cells. These proteinases also induce macrophage and airway epithelial cell‐based antifungal immunity 15. Among other actions these proteinases cleave fibrinogen, producing fibrinogen‐derived cleavage products (FCPs) that signal through Toll like receptor 4 to elicit antifungal immunity. They also promote asthma by upregulating mucin genes and the cytokine‐binding component of the IL‐13 receptor, IL‐13Rα1 15.

Many different proteinases (e.g., dust mite, fungal, etc.) are found in household dust, but these enzymes are either denatured and no longer exhibit proteinase activity or exist in levels insufficient to induce allergic inflammation and disease. Thus, it is unlikely that these proteinases have a direct role in asthma pathogenesis 16. On the other hand, there is evidence that asthma may be provoked by inhaling fungal conidia present in household dust 16. We and others have shown that fungi, particularly aspergillus species, which are abundant in ambient air, can infect the airways of normal mice. These infections generate sufficient aspergillopepsin and other proteinases within the lungs to induce asthma‐like disease 16, 17, 18, 19. Additional evidence supporting the hypothesis that fungi found in household samples are involved in the pathogenesis of asthma is the fact that these same fungi can be cultured from the airway secretions (sputum, sinus lavage) of subjects with asthma and chronic rhinosinusitis 18, 19, 20, 21. Moreover, specific immunity to fungi (fungus‐specific T_H_2 responses) can be demonstrated in many asthmatics, suggesting that rather than innocuous colonization, the airway fungi are producing an immunologically and physiologically significant infection that we term airway mycosis 20.

Multiple clinical trials have explored the possibility that suppressing or even eliminating airway mycosis with antifungals may improve asthmatics. For example in one study 32 weeks of treatment with itraconazole was associated with improvement in Asthma Quality of Life Questionnaire (AQLQ) scores, rhinitis scores, and peak airflow while simultaneously reducing total serum IgE levels 22. These findings, however, could not be replicated in a subsequent, similarly‐designed trial with the closely related agent voriconazole 23.

Given the positive result of one randomized clinical trial 22 and reports from several small case‐control studies and retrospective reviews 24, 25, 26, 27, 28, we retrospectively reviewed our clinical experience using a selected group of asthmatics with airway mycoses who had failed standard therapy; these patients were referred to the Allergy and Immunology service at the Michael E DeBakey VA Medical Center between 2012 and 2016. They were treated for 4–52 weeks predominantly with azole‐based antifungals. Their clinical response as measured by asthma control, PEFR, serum total IgE, and absolute blood eosinophil counts before and after antifungal therapy was assessed and compare to a control group who received only standard of care without antifungal therapy.

## Materials and Methods

### Study design and population

We conducted a retrospective chart review of 50 asthma patients referred to the Michael E. Debakey VA Medical Center Allergy & Immunology Clinic for refractory asthma between 2012 and 2016. All patients whose sputa grew fungi were offered antifungal therapy in addition to standard care. Forty‐one patients agreed to be treated with antifungals including terbinafine, voriconazole, itraconazole, fluconazole, or posaconazole for at least 4, and up to 52 weeks. Nine patients whose sputa contained fungi were treated with standard bronchodilators and anti‐inflammatory agents but did not receive antifungals because they did not tolerate these, refused antifungal therapy, or had medication incompatibilities. Chart review included patients with at least 1 follow up within 1 year of their initial visit. The retrospective chart review was approved by the Baylor College of Medicine and the VA Institutional Review Boards.

### Fungal culture and assessment of airway mycosis

Patients referred for moderate to severe asthma underwent routine evaluation for airway mycosis as previously reported by our group 20. Briefly, patients collected their first morning purulent sputum in sterile specimen cups, discarding specimens that were clear or colorless. Samples were kept frozen until returned to the investigators and were batch processed. Sputum samples were thawed at room temperature then diluted with 2 mL 1 M dithiothreitol (DTT) in 50 mL conical tubes. Sterile phosphate buffered saline (PBS) was then added to a total volume of 25 mL. The mixture was extensively vortexed at room temperature for 30 min and then placed in a 37°C water bath for 30 min and then centrifuged at 4,000 RPM (700 G) for 15 min. The supernatants were discarded and the pellets resuspended in 1 mL of sterile PBS, repeating mucolysis with DTT as needed for extremely viscous specimens. A total of 250 μL of the suspension were then spread onto two Sabouraud's culture plates; one was incubated at 37°C and one at 25°C. The plates were incubated for up to 3 weeks before final evaluation. Airway mycosis was defined by having positive sputum samples for fungal growth in any patient with asthma, with a positive sample defined as growing six or more distinct fungal colonies (mold or yeast).

### Antifungal therapy

Patients receiving a diagnosis of asthma with airway mycosis were treated with one of five antifungals, terbinafine, voriconazole, itraconazole, fluconazole, or posaconazole. Therapy was initiated using standard dosing for invasive fungal disease, adjusting the dose based on trough drug levels, when available. Treatment was continued for a minimum of 12 weeks when tolerated. Patients treated for at least 4 weeks were included in the analysis. Liver function was measured 2 weeks after the initiation of therapy. Patients were examined 12 weeks after initiating therapy except where disease severity dictated earlier and more frequent evaluation. In addition to obtaining a medical history focused on asthma symptoms and general well‐being, vital signs, oxygen saturation, and peak expiratory flows were measured at each clinic visit. Telephone calls were used to assess patient progress and adjust medication dosage based on drug levels or evidence of toxicity.

### Data collection

In addition to general demographics, we collected data on the types of fungi cultured (mold, yeast, or both), length of therapy, relapse rates, and antifungal therapy discontinuation data. To gain insight into the effectiveness of antifungal therapy, we estimated asthma severity and measured peak expiratory flow rates, serum total IgE, and absolute blood eosinophil counts before and after the initial course of therapy. Because smoking might modify treatment response, we analyzed data from never‐ and ever‐smoker subgroups separately. The same information was collected at the same intervals from the nine patients with airway mycoses that were not treated with antifungals.

### Outcome measures

The primary outcome measures of this study were (A) asthma control as defined by the National Heart, Lung and Blood Institute (NHLBI) analog assessment scale for asthma control: 1 = very poorly controlled; 2 = not well controlled; and 3 = well controlled 24, (B) peak expiratory flow rate (PEFR), (C) serum total IgE levels, and (D) absolute blood eosinophil counts.

Response to therapy was defined as improvement in any one or combination of the following subjective variables: improved breathing and/or decrease in cough, sputum production, wheezing, rescue inhaler use, anti‐inflammatory controller inhaler use, or decreased us of systemic steroids. These data were recorded 1–4 weeks prior to initiation of antifungal therapy and at the next clinic visit 4–12 weeks after initiating treatment.

Asthma relapse was defined as the reappearance of cough, productive sputum, shortness of breath, wheezing, or any combination of these symptoms up to 24 weeks after cessation of antifungal therapy.

### Statistical analysis

Comparisons of two sets of paired data were made using the Wilcoxon signed‐rank test or Mann–Whitney test, as appropriate, to determine clinical and immunological responses to antifungal treatment. A log‐rank test was performed to compare the relapse‐free probabilities between groups. All analyses were performed using Stata v11.1 software (StataCorp, College Station, TX, USA) or Prism v5.0.2 (GraphPad Software, San Diego, CA, USA). All *p* values were two‐sided, with *p* < 0.05 considered statistically significant.

## Results

We reviewed 50 patients whose sputum cultures contained fungi. All were on standard bronchodilator and anti‐inflammatory inhaler treatment at the time of referral to the Allergy and Asthma subspecialty clinic. Forty one of these patients received a minimum of 4 weeks antifungal therapy and had at least 1 follow up visit. Nine patients continued to receive standard care but were not treated with antifungals because of medication intolerance, incompatibility, or refusal to accept this treatment and were used as control group to the antifungal treated patients.

### Demographic characteristics of case cohort and sputum fungal isolation

Approximately half of our subjects were never smokers; the other half identified as former or active smokers (ever smokers). The mean age of the 50 patients was 59 years, 77% were male, and 54% were Caucasians. All patients received inhaled steroids and the majority received at least one course of oral steroids (Table 1). Sputum cultures yielded both hyphal fungi (molds) and yeast forms, with hyphal forms being slightly more predominant. Sputa from about 1/3 of patients contained both forms of fungi (Table 2). We did not speciate our cultures as we believe all positive results warrant consideration for antifungal treatment and the lack of data indicating the utility of tailored antifungal therapy based on speciation.

**Table 1:** Demographic characteristics of all subjects with airway mycosis and asthma

Numbers (*N*)	All (50)	Treated never smokers (26)	Treated ever smokers (15)	Untreated (9)
Age ± SD	59 ± 14	54 ± 18	61 ± 9	60 ± 15
Males (%)	40 (77)	20 (77)	15 (88)	5 (56)
FEV_1_ % predicted ± SD	71 ± 20	68 ± 22	71 ± 16	78 ± 21
FVC % predicted ± SD	69 ± 10	69 ± 11	69 ± 8	70 ± 6
Smoking, *N* (PPY ± SD)				
Total	18 (37 ± 20)	0 (N/A)	15 (38 ± 22)	3 (32 ± 13)
Active	4 (47.5 ± 25)	0 (N/A)	4 (47.5 ± 25)	0 (N/A)
Former	14 (34 ± 19)	0 (N/A)	11 (35 ± 21)	3 (32 ± 13)
Never	26 (N/A)	0 (N/A)	0 (N/A)	0 (N/A)
Asthma	50	26	15	9
COPD (%)	13 (25)	1 (0.04)^a^	7 (41)	5 (56)
Inhaled Steroid (%)	52 (100)	26 (100)	15 (100)	9 (100)
Oral Steroid (%)	41 (78)	20 (77)	14 (93)	7 (78)

^a^ Reported second hand smoke exposure.

**Table 2:** Fungi recovered from cultured sputum

	Never smokers (44)	Ever smokers (17)	No antifungal (31)	Total (92)
Hyphal (molds)	19	10	14	43
Yeast	16	7	6	29
Both	9	0	11	20

### Length of antifungal therapy and reduction in asthma symptoms

Many patients received more than one antifungal. The duration of treatment was controlled by the decisions of the treating physicians and VAMC pharmacy policies. Most patients including 14 never‐ and nine ever‐smokers were treated with voriconazole (Table 3). Approximately 79% of never‐smokers and 100% of ever‐smokers, a group that includes patients with significant COPD, treated with voriconazole, reported a subjective response to treatment. Data from the small numbers of subjects treated with other azole antifungals (fluconazole, itraconazole, and posaconazole) were pooled. Positive responses in this group were similar to that seen with voriconazole while among those treated with terbinafine, 56% reported any benefit (Table 3). Two patients in the ever‐smoker group were treated with other azoles, and one responded to treatment, while 71% of ever smokers treated with terbinafine reported improvement in symptoms (Table 3).

**Table 3:** Length of antifungal therapy and outcome in never and ever smoker asthmatic subjects with airway mycosis

	Never smoker		Ever smoker	
Antifungal	Number of cases	Length therapy mean wks (range)	Number of cases	Length therapy mean wks (range)
Voriconazole (*N*)	(14)		(9)	
Positive response^a^ (%)	11 (78.6)	14 (6‐28)	9 (100)	11 (4‐26)
No response^b^ (%)	3 (21.4)	11 (6‐12)	0 (0.0)	N/A
Fluc/Itra/Posa^c^ (*N*)	(11)		(2)	
Positive response (%)	7 (63.6)	14 (4‐12)	1 (50.0)	17 (17)
No response (%)	4 (36.4)	12 (12)	1 (50.0)	12(12)
Terbinafine (*N*)	(16)		(7)	
Positive response (%)	9 (56.3)	9 (6‐12)	5 (71.4)	8 (6‐12)
No response (%)	7 (43.8)	7 (6‐12)	2 (28.6)	13 (6‐12)

^a^ Patient improvement in any one or combination of the following variables: Decreased cough, improved breathing, decreased sputum, decreased wheezing, decreased rescue inhaler use, decreased controller use, decreased systemic steroid use.

^b^ No significant improvement in any of the clinically relevant responses describe in^(a)^.

^c^ Fluc: fluconazole; Itra: itraconazole; Posa: posaconazole.

### Early discontinuation of antifungal therapy

Discontinuation rates were highest for voriconazole (21.4%) in the never smoker group (Table 4), consistent with previously published discontinuation rates for this agent 25, 29, 30. Among other antifungal treatment groups, 1 patient out of 16 treated with terbinafine (6.3%) and 1 patient out of 11 treated with all other antifungals (9.1%) had to discontinue due to adverse effects (Table 4).

**Table 4:** Discontinuation rates of antifungal therapy in never and ever smoker asthmatic subjects with airway mycosis

	Never smoker		Ever smoker	
Antifungal	Number of cases	Length therapy mean wks (range)	Number of cases	Length therapy mean wks (range)
Voriconazole (*N*)	(14)		(9)	
Discontinued^a^ (%)	3 (21.4)	11 (8‐12)	0 (0.0)	N/A
Fluc/Itra/Posa^b^ (*N*)	(11)		(2)	
Discontinued (%)	1 (9.1)	6 (6)	0 (0.0)	N/A
Terbinafine (*N*)	(16)		(7)	
Discontinued (%)	1 (6.3)	4 (4)	0 (0.0)	N/A

^a^ Discontinued medication due to adverse effects including psychological disturbance in patients with history of posttraumatic stress disorder.

^b^ Fluc: fluconazole; Itra: itraconazole; Posa: posaconazole.

### Subjective changes in asthma control

**Figure 1:**
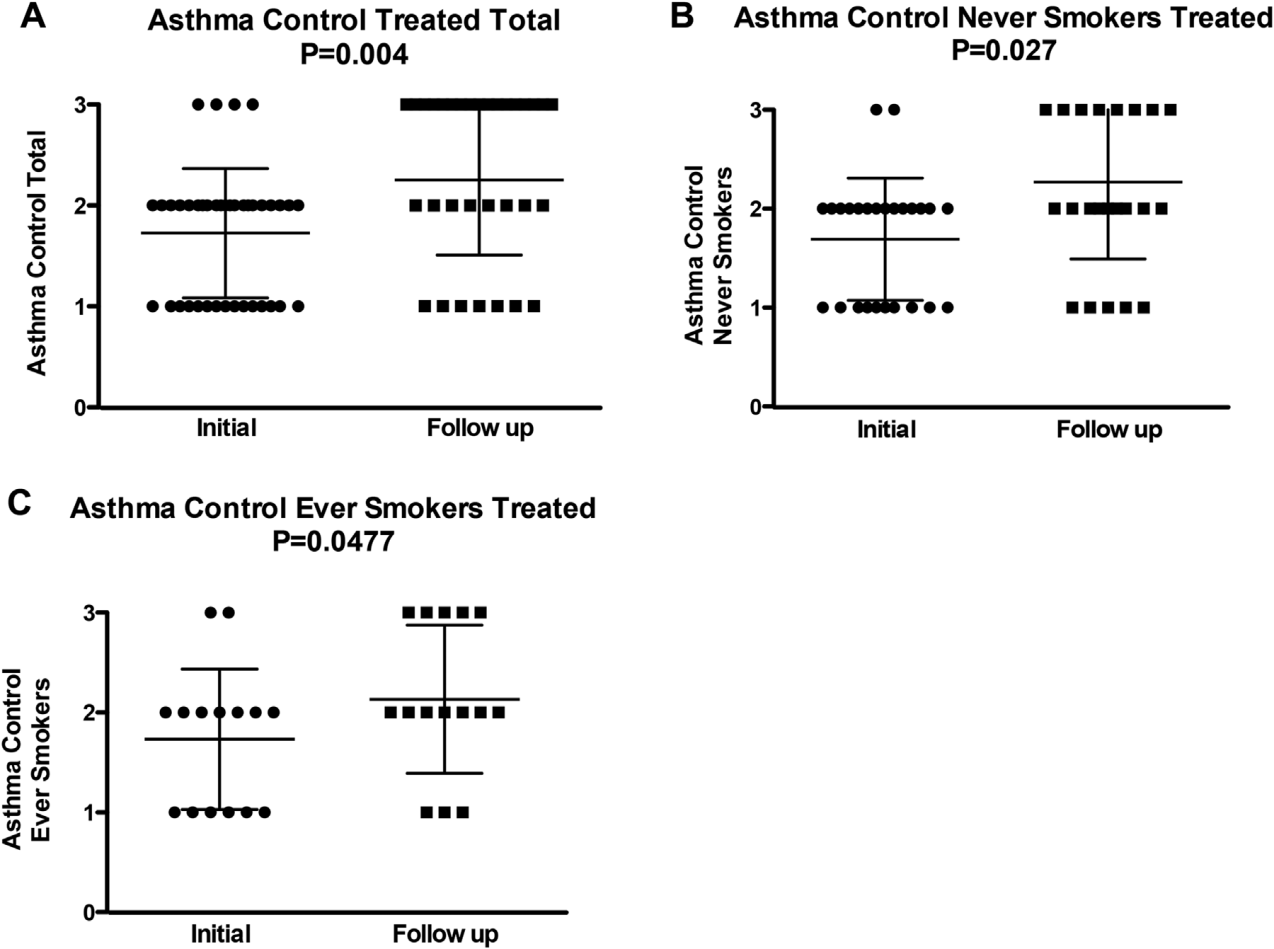
Asthma control before and after treatment with antifungals. Level of asthma control assessed using the analog scale based on the NHLBI assessment of asthma control (1 = very poorly controlled; 2 = not well controlled; and 3 = well controlled) was recorded 1–4 weeks prior to initiation of therapy (Initial) and next clinic visit (Follow up) 4–12 weeks after initial antifungal treatment in (A) total subjects (*N* = 41), (B) never‐ (*N* = 26) and (C) ever‐smokers (*N* = 15).

Asthma control improved in all treatment groups after initial antifungal therapy (1.72–2.25, *p* = 0.004) (Fig. 1A), including both never‐ and ever‐smokers (Fig. 1B and C). Several patients, however, required more than 4 weeks treatment and/or a change to another antifungal (data not shown). About one third of those reporting symptomatic improvement had significant improvement in peak expiratory flow values, and discontinued most or all asthma medications (data not shown).

### Objective changes in PEFR

**Figure 2:**
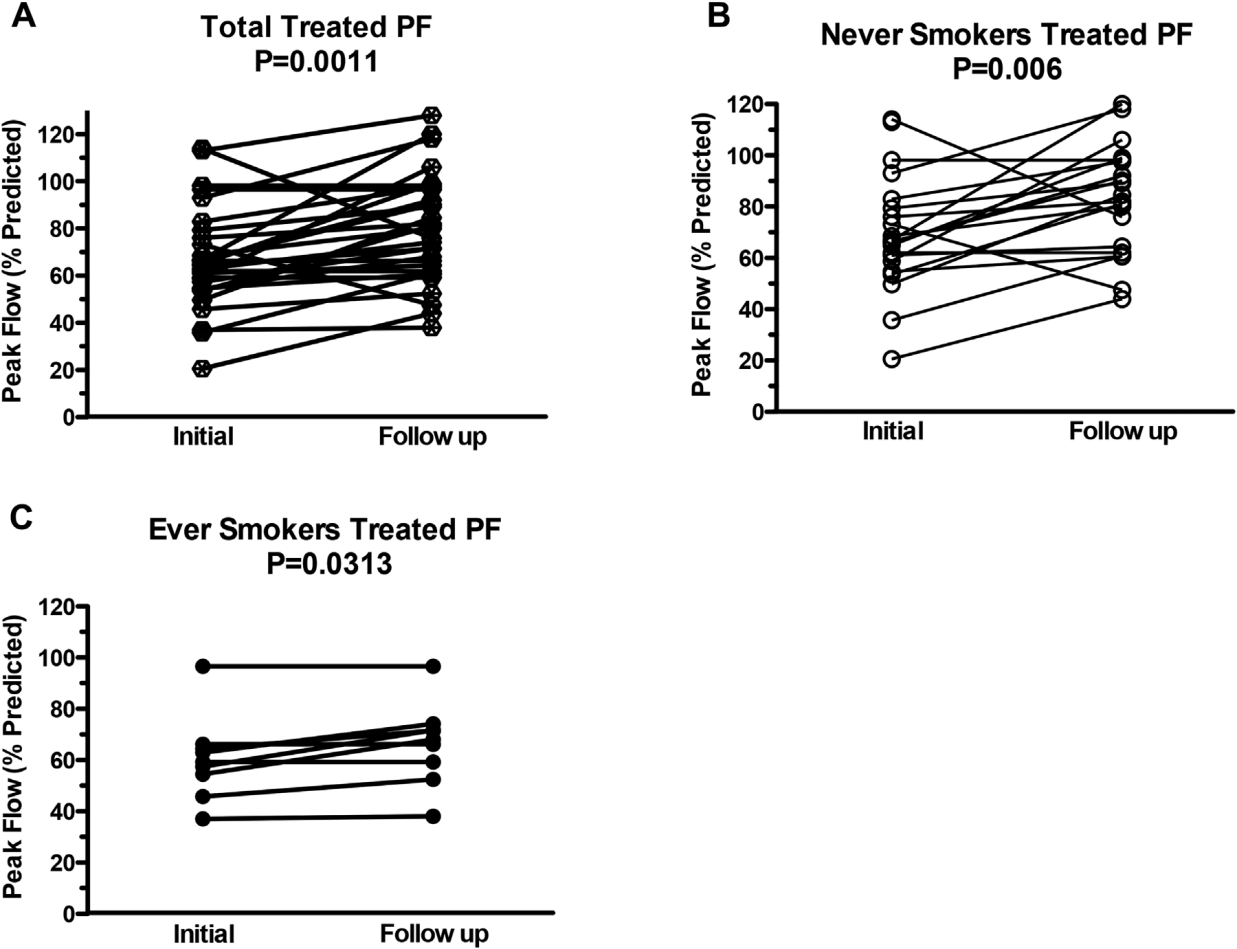
Change in peak flow (% predicted) following antifungal treatment. Paired PEFR (PF) measurements assessed in (A) total subjects (*N* = 30), (B) never smokers (*N* = 21) and (C) ever smokers (*N* = 9). PF measurements were recorded immediately prior to (Initial) and following 12 weeks of treatment (Follow up) and are presented as % predicted PEFR for each patient. Lines connect data before and after antifungal therapy for the same patients. Significance was calculated using the Wilcoxon signed‐rank test.

In treated patients percent predicted Peak Expiratory Flow Rates significantly improved from a pre‐treatment average of 69.4–79.3% after treatment (*p* = 0.0011, Fig. 2A). PEFR improved both in never‐ and ever‐smokers (Fig. 2B and C). Improvement in PEFR correlated with subjective symptomatic improvement (Fig. 1B and C).

### Systemic immune responses to antifungal therapy

**Figure 3:**
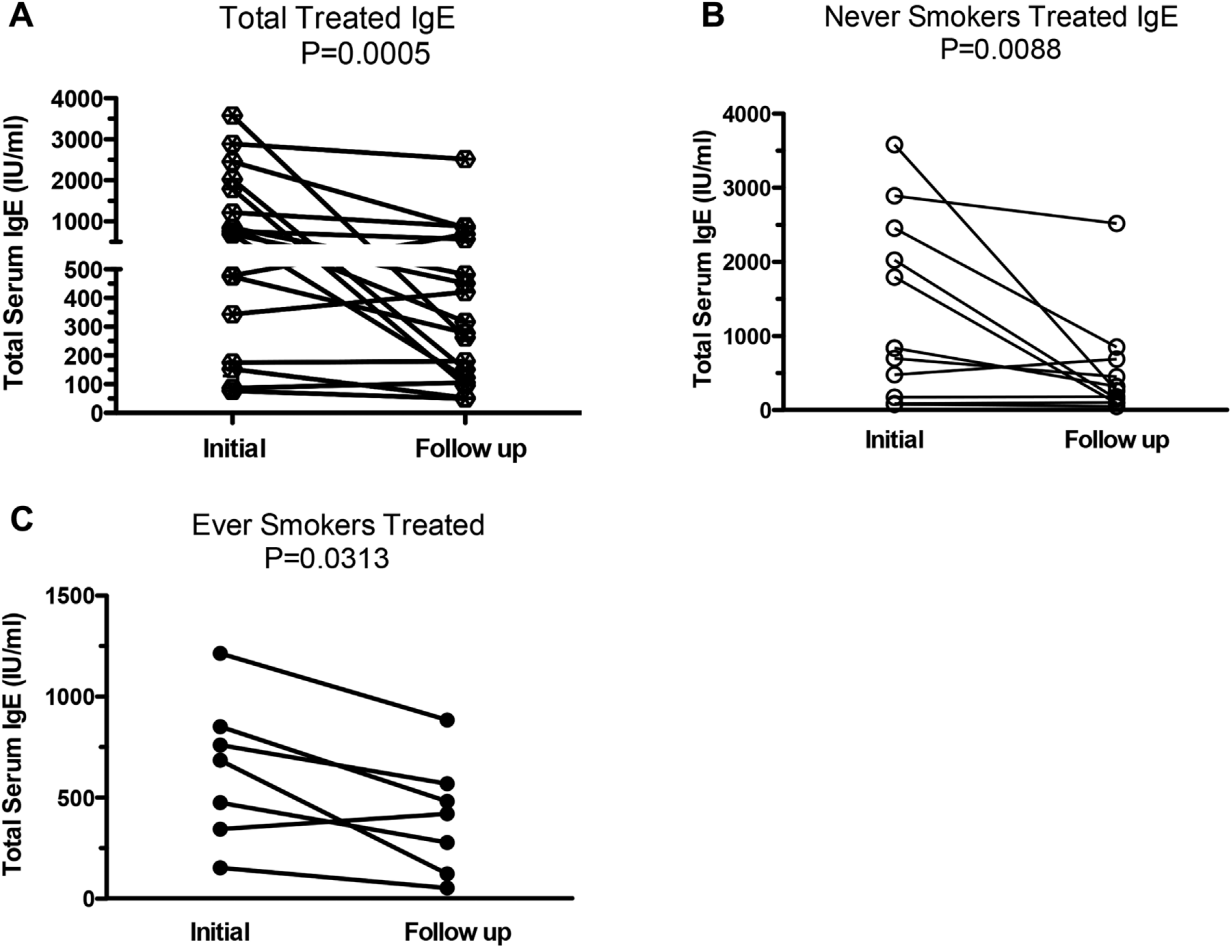
Changes in total IgE following antifungal treatment. Total IgE measurements assessed in (A) total subjects (*N* = 19), (B) never‐ (*N* = 12), and (C) ever smokers (*N* = 7). Measurements were recorded 4–16 weeks prior to initiation of therapy (Initial) and 4–16 weeks following (Follow up) initial antifungal therapy. Data are presented as absolute total serum IgE (IU/mL) for each patient with significance calculated using the Wilcoxon signed‐rank test. Lines connect data before and after antifungal therapy for the same patients.

**Figure 4:**
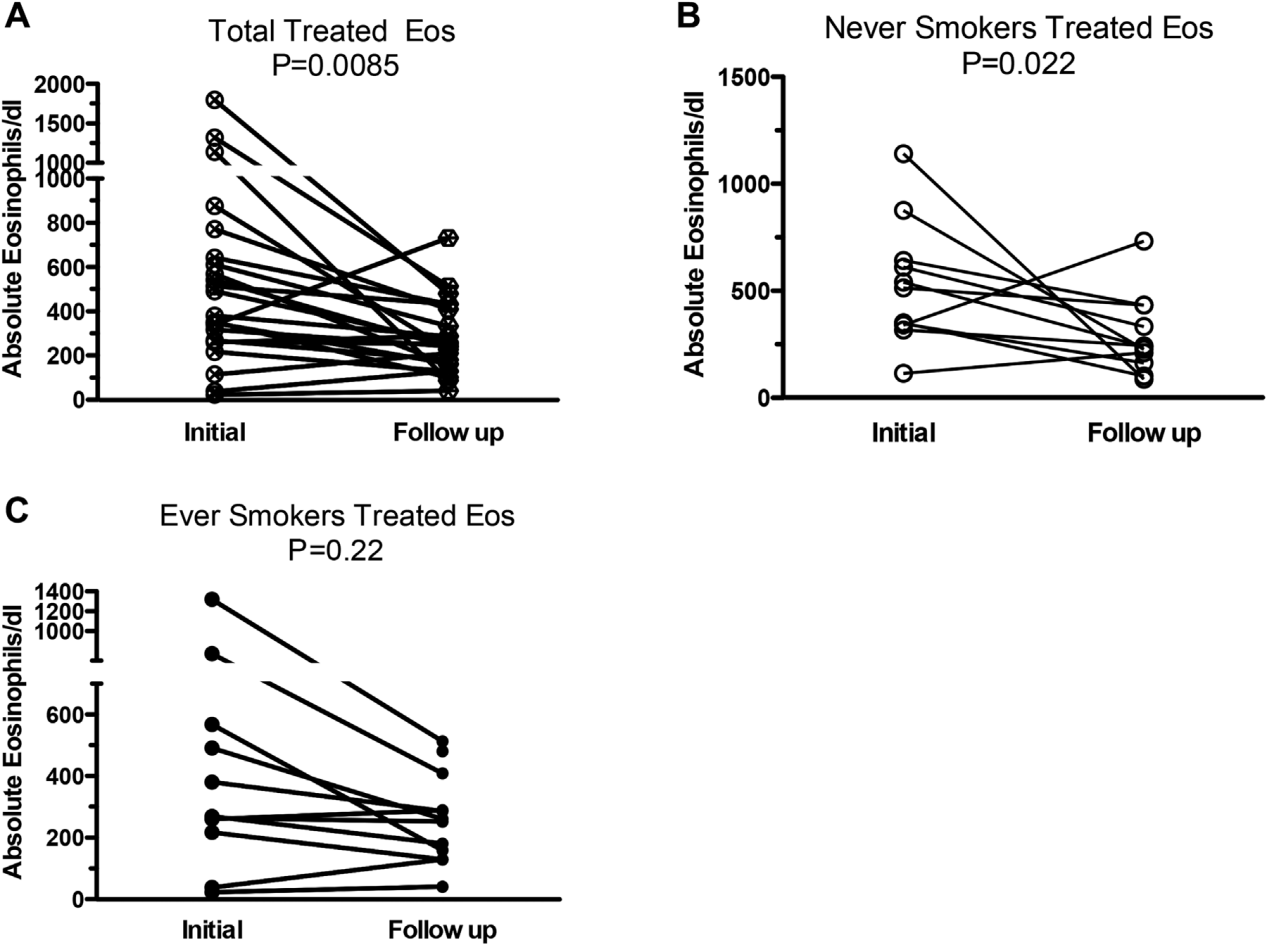
Changes in blood eosinophilia following antifungal treatment. Blood eosinophil measurements assessed in (A) total subjects (*N* = 23), (B) never‐ (*N* = 11), and (C) ever‐smokers (*N* = 12). Measurements were recorded 4–16 weeks prior to initiation of therapy (Initial) and 4–16 weeks following (Follow up) initial antifungal therapy. Lines connecting data before and after antifungal therapy for the same patients. Data are presented as total blood eosinophils/dL with significance calculated using the Wilcoxon signed‐rank test.

Treatment was associated with a significant reduction in total IgE levels (1,075–463 kU/L, *p* = 0.0005) (Fig. 3A), evident in both never‐ and ever‐smokers (Fig. 3B and C). In several never‐smokers serum IgE fell 10‐fold with treatment to levels within the normal range (Fig. 3B). Treatment was also associated with significant reductions in absolute blood eosinophil counts (*p* = 0.0095) (Fig. 4A). This fall was significant only in never‐smokers (Fig. 4B and C).

### Increased time to relapse in asthma symptoms with greater than 12 weeks of antifungal therapy

**Figure 5:**
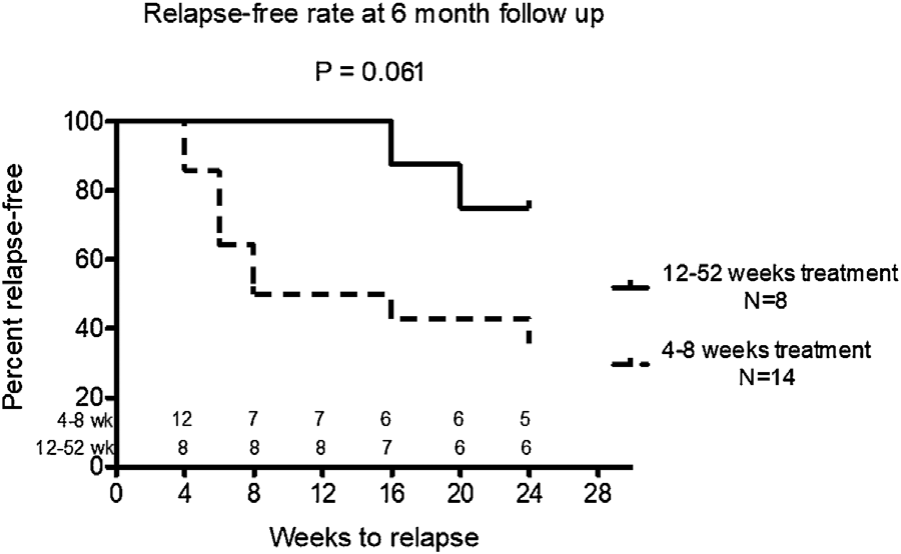
Time to relapse of asthma symptoms. Kaplan–Meier curve for asthma symptom recurrence within 6 months of initial antifungal based on duration of treatment (<8 weeks or >12 weeks). Outcomes of patients treated with azole‐based antifungals (itraconazole, fluconazole, voriconazole, or posaconazole) were examined to determine the number of asthma symptom recurrences based on the length of treatment. Disease recurrence was defined as recurrence of any asthma related symptom (cough, productive sputum, shortness of breath, wheezing) within six months of completion of antifungal therapy. *p* = 0.061, log‐rank test.

Because azole‐based antifungal therapy duration significantly varied in this asthmatic cohort, we next compared the relapse‐free probabilities in those who received less than 8 or greater than 12 weeks of antifungal therapy. A log‐rank analysis showed a trend for higher relapse‐free probability in patients who received a minimum of 12 weeks of antifungal therapy (*p* = 0.061) (Fig. 5).

### Asthmatics with airway mycoses not treated with anti‐fungal antibiotics

**Figure 6:**
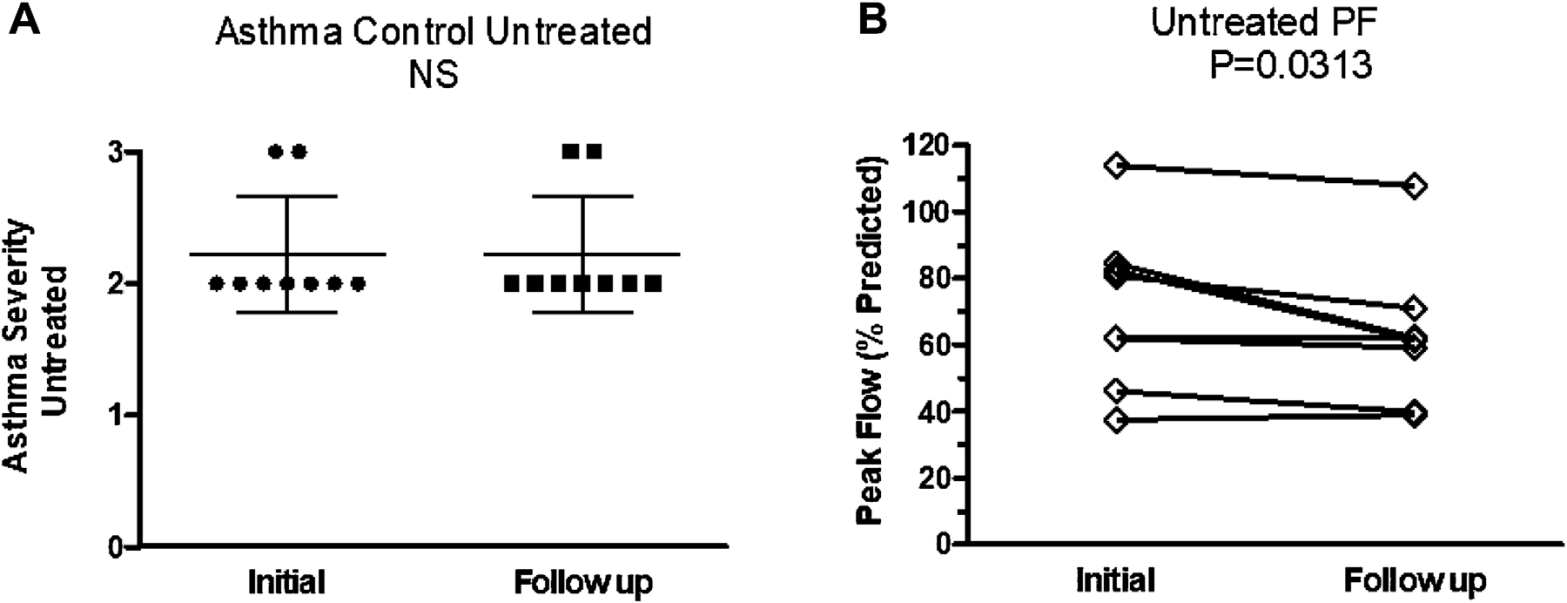
Asthma severity and peak flow changes in asthmatics with airway mycosis who were not treated with antifungals. (A) Asthma severity (*N* = 9) and (B) PEFR (peak flow; % predicted) (*N* = 9) were measured in asthmatic subjects with airway mycosis who were not treated with antifungals. Data are presented as means ± SEM (Asthma severity) or individually with lines connecting the same patient prior to and after 12 weeks of standard asthma therapy without antifungals. Significance was calculated using the Wilcoxon signed‐rank test.

We next assessed clinical follow up in nine asthmatics that we identified with airway mycosis who were referred to the Allergy and Immunology Clinic and given standard of care, but not treated with antifungals. The reasons for lack of antifungal therapy included intolerance, refusal, or medication incompatibility. Although these patients continued to receive standard asthma therapy (e.g., inhaled and/or oral steroids, long and short acting beta agonists), chart review of asthma severity using the same parameters described for the antifungal‐treated group showed no significant changes (Fig. 6A). Moreover, PEFR (% predicted) significantly worsened after 12 weeks of follow up (70.2–62.8, *p* = 0.0313; Fig. 6).

## Discussion

The patients referred for this study reported asthma symptoms for most of their adult lives. Despite close adherence, they had failed to respond to conventional therapy and often had progressive disease that required frequent, extended courses of systemic corticosteroids. In many, their significant symptomatic response to antifungal therapy was accompanied by reduced serum IgE, reduced peripheral eosinophil counts, and improved peak expiratory flow values regardless of smoking status. Thus, evidence of improvement in these treatment‐refractory patients is particularly noteworthy. Our novel methods for culturing fungi from sputum 31, which eliminates fungistatic mucins and enhances recovery and identification of living microorganisms, helped us identify patients in whom fungus infections contributed to the pathophysiology of their disease. We suggest that future randomized clinical trials that use this improved culture method will be more likely to identify patients with airway mycoses who can benefit from antifungals.

Although fungal infections have been implicated pathophysiologically in diverse allergic airway diseases 18, 20, 26, 31, 32, treatment with antifungals has not been unequivocally beneficial. Treatment with itraconazole reportedly improved asthma symptoms 22 prompting sporadic use of antifungals in others with positive fungal cultures 20, 21, 22, 25, but the lack of efficacy of voriconazole in asthmatics 23 called to question the value of these agents and prompted us to evaluate outcomes of similar asthmatics treated with antifungals in our clinic. The results of our analysis support the notion that antifungal treatment, especially with voriconazole, results in symptomatic improvement in some adult asthmatics with moderate to severe disease. In these patients antifungal therapy attenuated allergic inflammation as estimated from serum IgE levels and peripheral eosinophil counts. We did not assess the effect of treatment on airway mycosis; nevertheless we assume that treatment reduced fungal burden especially in those patients who stopped producing sputum during the course of antifungal therapy.

The study by Agbetile, et al. 23, that reported that voriconazole was ineffective in treating asthma included many smokers; their burden of intractable non‐asthma‐related airway obstruction may have obscured actions of any drug that simply reduced fungal burden and its associated inflammation, thereby explaining the difference between their study and ours. Studies of anti‐fungal therapy in allergic bronchopulmonary aspergillosis (ABPA) suggested that an important effect of anti‐fungal agents such as itraconazole in such infections is to reduce production of allergens and other molecules that promote the immunologic and inflammatory responses that result in asthma 33. Treatment in some cases may be sufficient to reduce but not eliminate the infection; in others the anti‐fungal agents may have a negligible effect on production of the microbial products that stimulate the host responses that we recognize as asthma. Consequently, treatment, especially short term treatment, may appear ineffective. It follows that in those many cases where the infection is not eradicated the beneficial effects of treatment with anti‐fungal agents may vary both as a function of duration as well as the intensity of the treatment and may depend on the fraction of fungal biomass that is destroyed by these anti‐microbial agents over time as well as the rate at which the organisms re‐grow once the treatment is stopped.

Early in the treatment of asthmatics when the patient is receiving both corticosteroids and azole antifungals it is important to recognize that both itraconazole and voriconazole inhibit the activity of the hepatic cytochrome P450 enzyme CYP3A4. A consequence of this inhibition is that the half‐lives of corticosteroids are prolonged, resulting in a more potent anti‐inflammatory steroid effect in patients treated both with azole antifungals and a corticosteroid 34. Asthma may be better controlled because the anti‐inflammatory effects of the corticosteroids are enhanced by treatment with these anti‐fungal agents 35, 36. But considering that corticosteroid therapy is immunosuppressive and promotes fungal overgrowth and dissemination 37, 38, 39, 40, ideally one needs to use anti‐fungal agents long enough to sufficiently reduce the fungal biomass and the production of pro‐inflammatory fungal products that the need for anti‐inflammatory corticosteroids will decrease. Indeed, with prolonged treatment many of our patients were able to reduce or eliminate use of corticosteroids suggesting that their general improvement as manifest by reduced sputum production and increased PEFR resulted from the decreased release of fungal allergens and pro‐inflammatory agents that induce host responses 41, 42 that result in asthma.

Prolonged therapy with anti‐fungal agents is key to their effectiveness. Our data indicate that the duration of therapy with azole‐class antifungals was an important predictor of medication response. Amongst azole antifungals, relapse was almost universal in patients treated for less than 8 weeks. In contrast, relapse occurred in less than 50% of patients when therapy was extended to 12 weeks and in less than a quarter of patients treated for longer than 16 weeks (Fig. 5). Thus, a possible reason for the failure of the aforementioned voriconazole asthma clinical trial, was that it lasted only 12 weeks 23. Patients were treated in the successful itraconazole clinical trial for 32 weeks 22. In addition to length of therapy, several other factors may contribute to disease relapse including re‐exposure to mold from contaminated household or work environments, partial immunodeficiency against fungal pathogens, or combinations of these factors. Further studies are required to identify optimal protocols for use of antifungals in asthma and the reasons for disease recurrence.

One potential confounding factor in our study is the fact that many of our patients were either steroid dependent or steroid resistant and continued to receive pulse doses of oral corticosteroids concurrent with antifungal treatment. Consequently, some objective improvements, especially peripheral eosinophil counts and peak flow increases, may be explained in part by steroid treatment. However, serum total IgE level is not known to be affected by corticosteroid use and its consistent downtrend in our treated patients again supports the concept that efficacy of antifungal therapy was related to diminished fungal burden. The failure of a similar group of asthmatics to demonstrate improvement in any regard while receiving standard therapy without antifungals further supports this conclusion.

In summary, our analysis shows that multiple parameters of asthma disease severity significantly improved in adults treated with one or more antifungal agents. In contrast, patients with similarly severe asthma treated with conventional bronchodilator and anti‐inflammatory agents, but not with antifungals did not improve and over time and had no significant improvement in PEFR. Our findings underscore the need for additional randomized controlled studies to determine the utility of antifungal therapy in asthma and related respiratory diseases.

## Conflicts of Interest

I testify on behalf of all co‐authors that our article submitted to Immunity Inflammation and Disease, Benefits of Antifungal Therapy in Asthma Patients with Airway Mycosis: A Retrospective Cohort Analysis: (i) This material has not been published in whole or in part elsewhere. (ii) The manuscript is not currently being considered for publication in another journal. (iii) All authors have been personally and actively involved in substantive work leading to the manuscript, and will hold themselves jointly and individually responsible for its content.
